# Adipose-derived CLEC3B promotes osteogenesis through a CLEC3B–IGFBP4-related paracrine axis associated with mitochondrial adaptation in postmenopausal osteoporosis

**DOI:** 10.1007/s10565-026-10223-x

**Published:** 2026-07-03

**Authors:** Zhihao Xia, Liangliang Wei, Jiajia Ji, Shaobo Wu, Yue Sun, Gaoyang Zong, Xiangyang Li, Yuxing Ye, Yan Zhang, Dageng Huang

**Affiliations:** 1 Department of Spine Surgery, Honghui Hospital, Xi’an Jiaotong University, Xi’an, 710054 Shaanxi China; 2 Department of Orthopaedics, The First Affiliated Hospital of Xi’an Jiaotong University, Xi’an, People’s Republic of China; 3 Center for Translational Medicine, The First Affiliated Hospital of Xi’an Jiaotong University, Xi’an, People’s Republic of China; 4 Honghui Hospital, Xi’an Jiaotong University, Xi’an, 710054 Shaanxi China; 5 Department of Orthopaedics, Lanzhou University Second Hospital, Lanzhou, 730030 Gansu China

**Keywords:** Postmenopausal osteoporosis, CLEC3B, Adipose-bone crosstalk, IGFBP4, Osteogenesis, Mitochondrial adaptation

## Abstract

Postmenopausal osteoporosis (PMOP) develops within a complex metabolic milieu, but the adipose-derived cues that regulate osteoblast function are not fully defined. Here, plasma ELISA analysis showed preferential elevation of circulating C-type lectin domain family 3 member B (CLEC3B) in the high-BMI PMOP subgroup. CLEC3B was enriched in adipose tissue and negatively regulated by estradiol. *In vivo*, adipose-targeted overexpression of *Clec3b* increased trabecular bone mass and promoted osteoblast-associated bone formation without markedly altering osteoclast-related parameters. To investigate the underlying mode of action, we used an adipocyte–osteoblast conditioned-medium model. Conditioned medium from *Clec3b*-overexpressing adipocytes promoted osteogenic differentiation of primary calvarial osteoblasts, whereas conditioned medium from *Clec3b*-silenced adipocytes produced the opposite effect. Adipocyte transcriptomic analysis nominated Igfbp4 as a secretome-associated candidate reduced after *Clec3b* overexpression. Recombinant insulin-like growth factor-binding protein 4 (IGFBP4) reduced the increases in osteogenic gene expression, RUNX2 abundance, and matrix deposition induced by *Clec3b*-enriched conditioned medium. In parallel, recombinant IGFBP4 (rIGFBP4) partially attenuated the associated changes in mitochondrial superoxide-related fluorescence, intracellular oxidative fluorescence, mitochondrial membrane potential, and PINK1/PRKN-related signaling. Collectively, the data are consistent with a model whereby adipose-derived CLEC3B promotes osteogenesis through a CLEC3B–IGFBP4-related paracrine axis associated with mitochondrial adaptation in osteoblasts. These findings define a testable adipose-to-bone signaling mechanism relevant to osteoblast dysfunction in PMOP.

**Graphical Abstract:**

Proposed adipose-to-bone CLEC3B–IGFBP4-related paracrine axis associated with mitochondrial adaptation in osteoblasts.

• Plasma CLEC3B is preferentially elevated in high-BMI PMOP.

• Adipose-targeted *Clec3b* overexpression promotes trabecular bone accrual.

• Adipocytic *Clec3b* overexpression suppresses IGFBP4 and enhances osteogenic differentiation.

• The CLEC3B–IGFBP4-related axis is associated with mitochondrial adaptation in osteoblasts.

**Figure:**
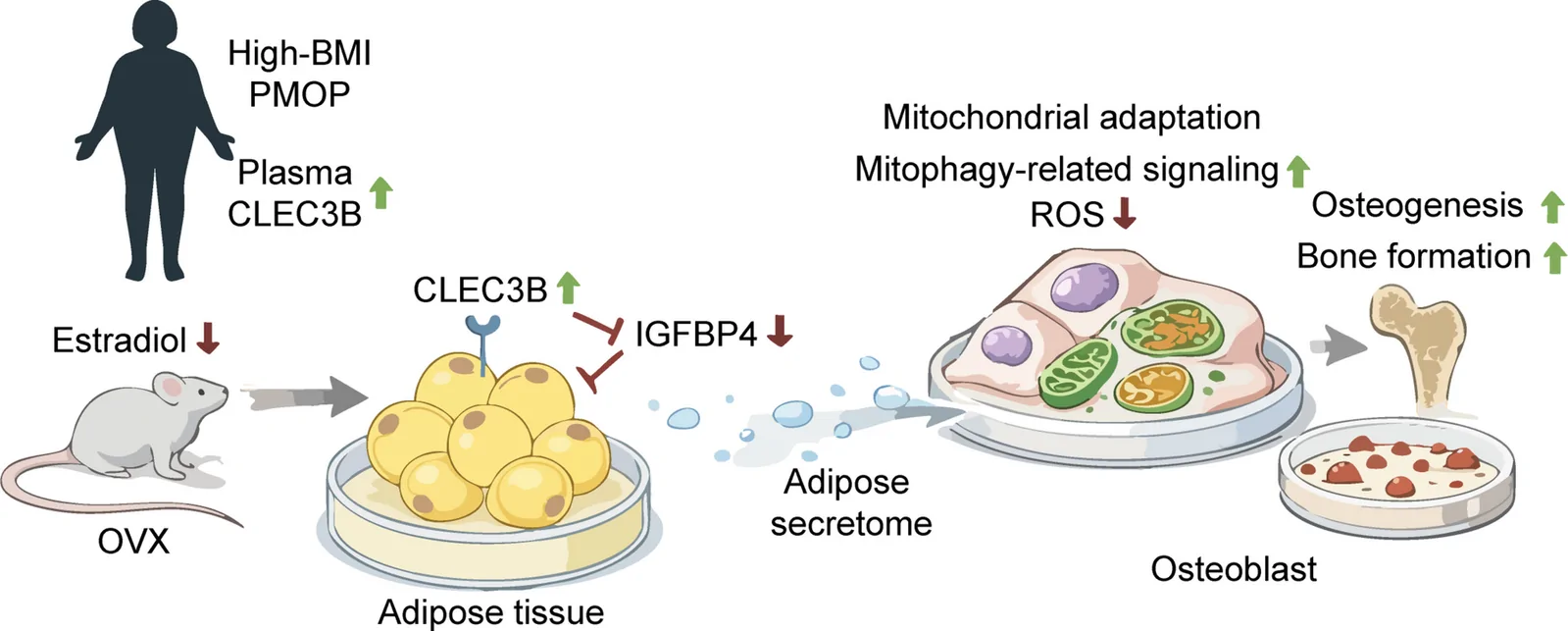

**Supplementary Information:**

The online version contains supplementary material available at https://doi.org/10.1007/s10565-026-10223-x.

## Introduction

Postmenopausal osteoporosis (PMOP) represents a key contributor to skeletal fragility in aging women, largely arising from estrogen depletion–mediated disruption of bone remodeling, in which bone formation fails to compensate for sustained resorption (Yao et al. 2025; Forte et al. 2023). However, the marked clinical heterogeneity of PMOP indicates that its pathogenesis cannot be explained by hormonal withdrawal alone (Hart 2024). Aging and menopause are frequently accompanied by increased adiposity and systemic metabolic dysfunction, yet the skeletal impact of obesity remains paradoxical (Juppi et al. 2022). Although increased mechanical loading may favor bone mass, obesity does not uniformly exert protective effects on the skeleton (Silva et al. 2019). Obesity-related inflammation, altered adipokine signaling, metabolic dysfunction, and adipose-tissue remodeling may impair bone homeostasis and osteoblast function, thereby contributing to heterogeneous skeletal outcomes (Forte et al. 2023). These observations suggest that metabolically responsive regulators linking adipose dysfunction to impaired osteogenesis may play underappreciated roles in PMOP pathogenesis and represent potential therapeutic targets (Paula and Rosen 2020).

Adipose tissue is increasingly viewed as an endocrine compartment rather than simply a site of energy storage (Ahima and Flier 2000). Beyond lipid storage, adipose depots release diverse bioactive mediators, including adipokines, cytokines, growth factor-binding proteins, free fatty acid–related signals, and extracellular vesicle cargoes, thereby affecting remote tissues such as liver, muscle, vasculature, and bone (Paula and Rosen 2020; Ahima and Flier 2000; Liu et al. 2023). Leptin suppresses bone formation through central hypothalamic and sympathetic pathways (Ducy et al. 2000; Takeda et al. 2002), whereas stromal cells expressing the leptin receptor also contribute substantially to adult bone-forming cells in the marrow niche (Zhou et al. 2014). Adiponectin has been reported to promote osteogenic differentiation, but its skeletal actions vary with systemic and local context (Liu et al. 2023). During obesity-related metabolic stress, adipose depots can acquire an inflammatory phenotype and release a remodeled secretome that includes interleukin-6 (IL-6), tumor necrosis factor-α (TNF-α), free fatty acid–related signals, and extracellular vesicles, with potential consequences for bone homeostasis (Ali et al. 2022; Musso et al. 2013; Zhang et al. 2021; Zhu et al. 2021). Together, these findings indicate that adipose–bone crosstalk is governed by a dynamic endocrine network shaped by hormonal status, inflammatory tone, and metabolic state rather than by a single adipokine. However, the circulating proteins linking adipose remodeling to impaired osteogenesis in PMOP remain poorly defined. Defining these mediators may clarify why increased adiposity and impaired skeletal homeostasis can occur together in some postmenopausal individuals (Veldhuis-Vlug and Rosen 2018).

Osteoblast differentiation and matrix mineralization are energy-dependent processes that require sustained mitochondrial function and redox balance (Veldhuis-Vlug and Rosen 2017; Lee et al. 2022; Gastel and Carmeliet 2021; Shen et al. 2022; Suh and Lee 2024). Under aging or metabolic stress, damaged mitochondria accumulate, adenosine triphosphate (ATP) production declines, and reactive oxygen species (ROS) increase, thereby impairing osteoblast survival and function (Nandy et al. 2024; Li et al. 2017; Lin et al. 2022). Accordingly, processes that preserve mitochondrial integrity, including mitophagy, are likely to support osteoblast fitness (Palikaras et al. 2015). In particular, PINK1/PRKN-associated signaling has been associated with osteoblast differentiation and stress-related bone formation (Wang et al. 2020). Nevertheless, how adipose-derived endocrine cues intersect with mitochondrial quality-control-related processes in osteoblasts remains incompletely understood (Li et al. 2023).

C-type lectin domain family 3 member B (CLEC3B), also known as tetranectin, is a secreted C-type lectin associated with extracellular matrix remodeling and tissue homeostasis (Holtet et al. 1997). Emerging evidence suggests that CLEC3B is an adipocyte-enriched secretory factor with potential endocrine relevance in metabolic disease and possible involvement in adipose–bone crosstalk (Liu et al. 2022; Wu et al. 2024; Huang et al. 2020). Building on our previous plasma proteomic profiling in PMOP (Huang et al. 2020), we selected CLEC3B for validation as a circulating candidate across subgroups defined by BMI and PMOP status. Transcriptomic analysis further highlighted insulin-like growth factor-binding protein 4 (IGFBP4) as a responsive node. Given the established role of IGFBP4 in bone remodeling (Maridas et al. 2017; Durham et al. 1994), we hypothesized that adipose-derived CLEC3B may remodel the adipocyte secretory program and alter IGFBP4-related paracrine signaling, thereby influencing mitochondrial adaptation and osteogenic differentiation in osteoblast-lineage cells. Here, we examined the skeletal role of adipose-derived CLEC3B in PMOP and assessed whether a CLEC3B–IGFBP4-related axis is associated with osteogenesis and mitochondrial adaptation in osteoblasts (Zhang et al. 2003; Wu et al. 2017).

## Materials and methods

### Clinical sample collection

Postmenopausal women were categorized by bone mineral density (BMD) T-scores measured using dual-energy X-ray absorptiometry (DXA). Participants with a T-score ≤ − 2.5 at L1–L4 or at the femoral neck were classified as PMOP, whereas those with a T-score > − 2.5 were classified as non-PMOP. Body mass index (BMI) was then used for secondary stratification into normal-BMI (< 25.0 kg/m^2^) and high-BMI (≥ 25.0 kg/m^2^) subgroups. Fasting venous blood was obtained, processed within 30 min, and stored as plasma aliquots at −80 °C.

### Measurement of plasma CLEC3B levels

Plasma proteomic profiling had been performed previously in a PMOP discovery cohort (Huang et al. 2020). In the present independent validation cohort, circulating CLEC3B was measured by enzyme-linked immunosorbent assay (ELISA) and analyzed according to BMI and BMD status. EDTA plasma samples were assayed with a Human Tetranectin (CLEC3B) ELISA Kit (CUSABIO, Wuhan, China). Concentrations were derived from the standard curve and reported as ng/mL. Clinical information and plasma CLEC3B values are provided in Supplementary Table S1.

### Publicly available human tissue-expression data

Human *CLEC3B* tissue-level RNA expression was obtained from the GTEx RNA-seq dataset through the Human Protein Atlas (HPA) portal (https://www.proteinatlas.org/ENSG00000163815-CLEC3B/tissue; accessed June 2, 2026). Values were downloaded as nTPM from the HPA tissue interface without further transformation. The HPA Tissue resource summarizes GTEx gene-level RNA-seq data across 36 tissues and 46 tissue subtypes.

### Animal models and adipose-targeted adeno-associated virus (AAV) delivery

10-week-old female C57BL/6 J mice were subjected to bilateral ovariectomy (OVX) or sham operation, and tissues were collected 8 weeks thereafter. For adipose-targeted gain-of-function experiments, 10-week-old female Adipoq-Cre mice received bilateral gWAT injections of AAV9-CAG-DIO-Clec3b or control vector. Each mouse received 5 × 10⁹ vector genomes in 50 μL total volume and was analyzed 8 weeks after injection.

### Culture, differentiation, and genetic manipulation of 3T3-L1 cells

3T3-L1 preadipocytes were cultured in growth medium. At 2 days post-confluence, adipogenic differentiation was initiated using DMEM/F12 (1:1) supplemented with 10% fetal bovine serum, (Sigma-Aldrich, St. Louis, MO, USA; 1.5 μg/mL insulin), 3-isobutyl-1-methylxanthine (Sigma-Aldrich; 0.5 mM), dexamethasone (Sigma-Aldrich; 1 μM), and, when required, rosiglitazone (Sigma-Aldrich; 1 μM). Following the induction period, cells were transferred to insulin-supplemented DMEM/F12 containing 10% fetal bovine serum and maintained until day 14, with medium changed every 2–3 days.

For estrogen-response experiments, differentiated 3T3-L1 adipocytes were treated with estradiol (MedChemExpress, Monmouth Junction, NJ, USA; 0, 1, 10, 50, or 100 nM) for 48 h. Full-length murine *Clec3b* was introduced using retroviral particles generated in Plat-E packaging cells, whereas *Clec3b* knockdown used lentiviral particles encoding a *Clec3b*-targeting shRNA. Transduction efficiency was confirmed using quantitative real-time PCR; vector information and the shRNA sequence are provided in the Supplementary Methods.

### Isolation and culture of primary bone-derived cells

BMSCs and BMMs were prepared from the femoral and tibial bone marrow of 8-week-old mice. The adherent fraction was expanded as BMSCs, whereas non-adherent cells were maintained in the presence of M-CSF to generate BMMs. Primary calvarial osteoblasts were obtained from calvariae of 3–5-day-old neonatal mice. Briefly, calvariae were cleaned, minced, and subjected to sequential digestion with collagenase type II (Sigma-Aldrich; 1 mg/mL) at 37 °C. After removal of the first digestion fraction, cells collected from the remaining fractions were pooled and cultured in α-MEM supplemented with 10% FBS and 1% penicillin–streptomycin at 37 °C in 5% CO₂.

### Preparation of conditioned media and recombinant IGFBP4 rescue experiments

For gWAT-derived conditioned medium (gWAT-CM), freshly collected gWAT from AAV-CT or AAV-Clec3b mice was minced, washed with phosphate-buffered saline (PBS), and maintained in α-MEM for 48 h. For 3T3-L1 adipocyte-conditioned medium (3T3-L1-CM), genetically modified mature adipocytes were washed and incubated in fresh α-MEM for 48 h. Supernatants were clarified by centrifugation at 1,000 × g for 10 min, passed through 0.22-μm filters, dispensed into aliquots, and kept at −80 °C.

For cell treatments, gWAT-CM or 3T3-L1-CM was diluted 1:2 with fresh medium and applied to BMSCs or primary calvarial osteoblasts, respectively. For rescue assays, recombinant murine IGFBP4 (rIGFBP4; R&D Systems, Minneapolis, MN, USA; 200 ng/mL) was added to 3T3-L1-CM-treated primary calvarial osteoblasts for 48 h in short-term assays or throughout osteogenic induction. To assess autophagy-related LC3B dynamics, chloroquine diphosphate salt (CQ; Sigma-Aldrich; 100 μM) was added during the final 3 h before protein collection, and LC3B-I/II levels were examined by western blotting.

### Osteogenic and osteoclast differentiation assays

Osteogenic induction was performed in α-MEM containing 10% fetal bovine serum, 50 μg/mL L-ascorbic acid, and 10 mM β-glycerophosphate. Alizarin Red S staining was performed after 18 days for BMSCs and 21 days for primary calvarial osteoblasts using 2% Alizarin Red S (Sigma-Aldrich), and mineralized area was quantified with ImageJ. Osteogenic markers (*Runx2*, *Alpl*, *Col1a1*, *Bglap*, *Spp1*) were measured by quantitative real-time PCR.

For osteoclast induction, BMMs were cultured with 30 ng/mL M-CSF and 100 ng/mL recombinant murine receptor activator of nuclear factor-κB ligand (RANKL; PeproTech), with medium changes every 2–3 days. After 5–7 days, osteoclasts were identified using a TRAP staining kit (Cosmo Bio Co., Ltd., Tokyo, Japan), and TRAP-positive cells with at least three nuclei were counted. F-actin rings were assessed after fixation, 0.2% Triton X-100 permeabilization, and Alexa Fluor 488-phalloidin staining (Invitrogen). Osteoclast-related genes (*Acp5*, *Ctsk*, *Nfatc1*, *Dcstamp*, *Itgb3*) were measured by quantitative real-time PCR. Primer sequences are listed in Supplementary Table S2.

### Alkaline phosphatase (ALP) staining and enzymatic activity assay

ALP staining and activity measurements were conducted in parallel 24-well plates after 10 days of osteogenic induction. For the staining procedure, cells were washed with PBS, fixed with 4% paraformaldehyde for 10 min, rinsed, and incubated with BCIP/NBT staining solution (Beyotime, Shanghai, China). Whole-well images were acquired under identical settings. ALP activity was quantified with a Nanjing Jiancheng assay kit and normalized to total protein.

### Micro-computed tomography (micro-CT)

Femora were fixed with 10% neutral-buffered formalin and subjected to scanning using an eXplore Locus SP micro-CT system (GE Healthcare). Trabecular parameters were quantified within a region extending proximally from the distal growth plate and corresponding to 20% of the femoral length, while cortical parameters were assessed at the mid-diaphyseal level. The evaluated indices comprised BMD, BV/TV, Tb.N, Tb.Th, Tb.Sp, Ct.Th, and Ct.Ar.

### Histology and immunostaining

Femora were decalcified in 0.5 M EDTA, paraffin-embedded, sectioned at 5 μm, and subjected to H&E, TRAP, RUNX2, NFATC1, and CLEC3B staining using the indicated commercial kits or antibodies. gWAT and additional tissues (heart, liver, kidney, femur, and BAT) were fixed in 4% paraformaldehyde and processed for CLEC3B immunofluorescence. Antibody information is listed in Supplementary Table S3.

### RNA extraction and RT-qPCR

Total RNA was isolated using TRIzol and converted to cDNA with a PrimeScript kit. qPCR amplification was conducted with TB Green chemistry on a CFX96 system, and relative gene expression was determined using the 2^ − ΔΔCt method with *Actb* as the internal reference gene.

### Western blotting

Protein concentrations in lysates were measured using a BCA assay. Equal amounts of protein were resolved by SDS–PAGE, electrotransferred onto PVDF membranes, and probed with the specified primary antibodies. Signals were visualized by chemiluminescence and analyzed with ImageJ. Antibody information is provided in Supplementary Table S3.

### RNA sequencing and bioinformatics analyses

Transcriptome sequencing of control and *Clec3b*-overexpressing 3T3-L1 adipocytes was conducted by Gene Denovo Biotechnology using the Illumina HiSeq 2500 platform. After quality filtering, clean reads were mapped to the reference genome and assembled with StringTie, followed by differential expression analysis using DESeq2. Differentially expressed genes were identified using thresholds of FDR < 0.05 and |log2 fold change|≥ 1.

### Mitochondrial morphology and oxidative stress analyses

For ultrastructural analysis, primary calvarial osteoblasts were fixed in 3% glutaraldehyde, post-fixed with 1% osmium tetroxide, embedded in Epoxy 812, and examined using a JEM-1400-SH transmission electron microscope. Mitochondrial length and mitochondria-ER contact (MERC) length were measured from TEM images. Mitochondria–lysosome colocalization was evaluated after a 30-min dual staining with MitoTracker Red CMXRos (Invitrogen; 100 nM) and LysoTracker Green DND-26 (Invitrogen; 50 nM). MitoSOX Red (Invitrogen; 500 nM) was used for 30 min at 37 °C, and DCFH-DA (Beyotime; 10 μM) or DHE (Beyotime; 5 μM) was applied for 20 min at 37 °C under light-protected conditions. JC-1 staining (Beyotime) was performed to evaluate mitochondrial membrane potential, which was expressed as the red/green fluorescence ratio. Fluorescence intensity was quantified using ImageJ.

### Image quantification and statistical analysis

Pearson correlation analysis was used to assess the association between BMI and plasma CLEC3B concentration. An exploratory multivariable linear regression model further tested this association after adjustment for age and PMOP status, with plasma CLEC3B entered as the outcome variable and BMI, age, and PMOP status entered as explanatory variables. The exploratory multivariable linear regression analysis was performed using R version 4.4.1. PMOP status was coded as 0 for non-PMOP and 1 for PMOP. Images were quantified in ImageJ/Fiji. Quantitative results are presented as mean ± SD, as indicated in the figure legends. Statistical testing was conducted in GraphPad Prism 9.0 using an unpaired two-tailed Student’s t-test or one-way ANOVA with Tukey’s post hoc test. A value of *p* < 0.05 was considered statistically significant, and n indicates biological replicates.

## Results

### Adipose-derived CLEC3B is preferentially elevated in the high-BMI PMOP subgroup and negatively regulated by estradiol

**Fig. 1:**
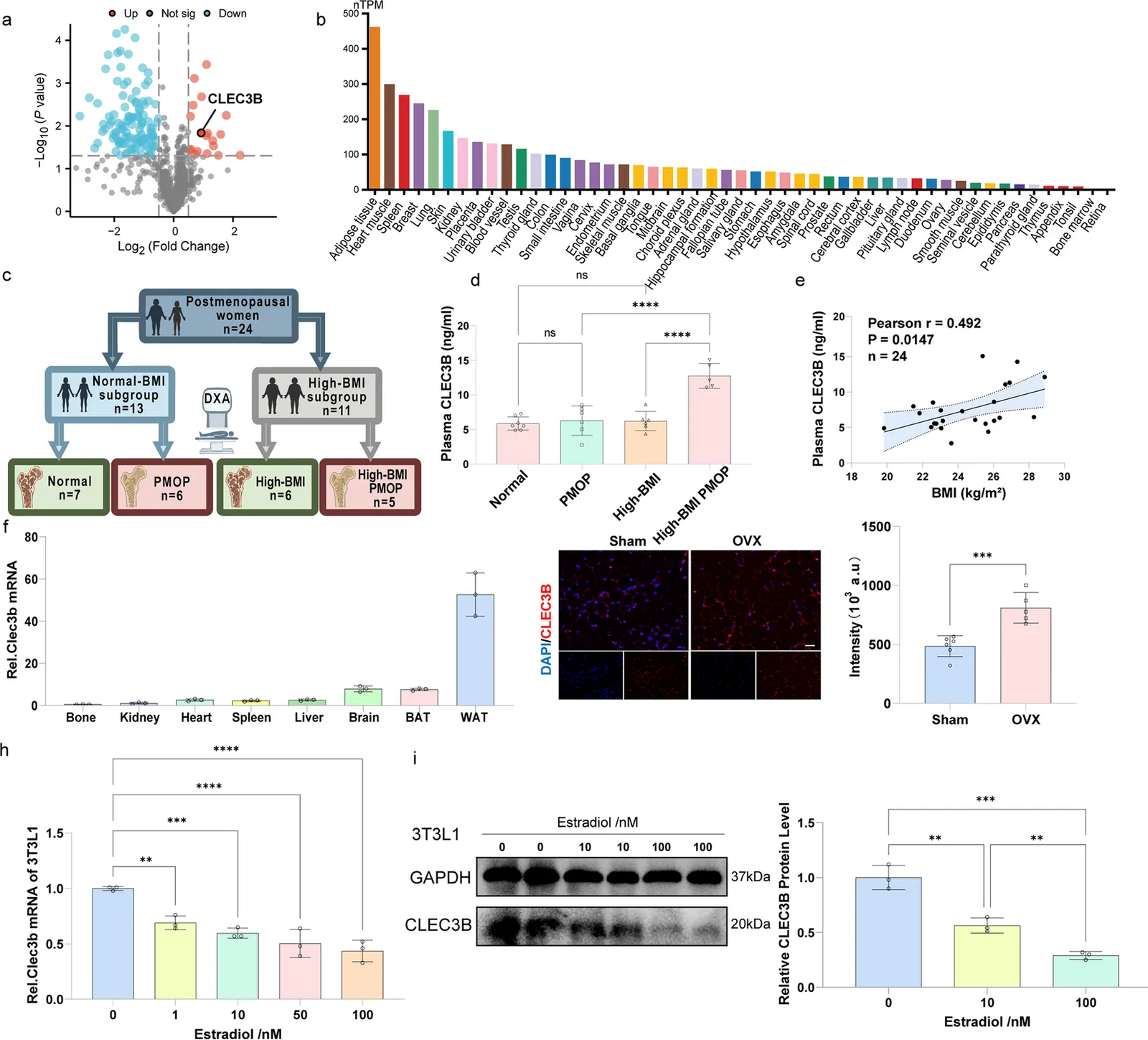
Adipose-derived CLEC3B is preferentially elevated in the high-BMI PMOP subgroup and negatively regulated by estradiol. **a** Volcano plot of the previously published plasma proteomic dataset showing differentially abundant proteins between participants with PMOP and controls. CLEC3B is highlighted as a differentially abundant protein. **b** Tissue-level RNA expression of human *CLEC3B* across tissues based on GTEx RNA-seq data accessed through the Human Protein Atlas portal. Expression values are reported as normalized protein-coding transcripts per million (nTPM) and represent the mean expression levels across individual samples within each tissue. GTEx gene-level RNA-seq data in the HPA Tissue resource cover 36 tissues based on 46 tissue subtypes. **c** Study design of the clinical cohort stratified according to BMI and PMOP status. For concise figure labeling, “Normal”, “PMOP”, “High-BMI”, and “High-BMI PMOP” denote the normal-BMI non-PMOP, normal-BMI PMOP, high-BMI non-PMOP, and high-BMI PMOP subgroups, respectively. **d** Plasma CLEC3B concentrations measured by ELISA in postmenopausal women stratified according to BMI and PMOP status. Circulating CLEC3B elevation was most prominent in the high-BMI PMOP subgroup. **e** Unadjusted Pearson correlation analysis showing a positive association between BMI and plasma CLEC3B concentration in the clinical cohort (*n* = 24). **f** qPCR analysis of *Clec3b* mRNA in various mouse organs (*n* = 3 per group). **g** Immunofluorescence staining of gWAT in Sham and OVX mice. Scale bar = 100 μm; CLEC3B signal (red) is increased in OVX (*n* = 6 per group). **h** Dose-dependent response of *Clec3b* mRNA in 3T3-L1 adipocytes treated with estradiol (n = 3 per group). **i** Corresponding western blot analysis of CLEC3B protein in 3T3-L1 cells (*n* = 3 per group). Data are shown as mean ± SD. *p < 0.05, **p < 0.01, ***p < 0.001, ****p < 0.0001. Statistical analyses were performed using two-tailed Student’s t-test or one-way ANOVA followed by Tukey’s post hoc test

Given the metabolic heterogeneity of postmenopausal osteoporosis (PMOP), we first sought to identify circulating factors potentially associated with distinct metabolic contexts of the disease. Re-examination of our previous plasma proteomic dataset showed higher CLEC3B abundance in PMOP patients than in non-PMOP (Fig. 1a). GTEx RNA-seq tissue-expression data obtained through the Human Protein Atlas portal further indicated that human *CLEC3B* was most abundant in adipose tissue among the analyzed tissues, consistent with its potential relevance as an adipose-enriched circulating factor (Fig. 1b). To further examine the clinical association between circulating CLEC3B, BMI, and PMOP status, we analyzed plasma CLEC3B concentrations by ELISA in an independent validation cohort of 24 postmenopausal women. Participants were stratified into normal-BMI non-PMOP, normal-BMI PMOP, high-BMI non-PMOP, and high-BMI PMOP subgroups (Fig. 1c). Plasma CLEC3B concentrations were markedly increased in the high-BMI PMOP subgroup, whereas no significant elevation was observed in either the normal-BMI PMOP subgroup or the high-BMI non-PMOP subgroup (Fig. 1d). Pearson analysis also indicated a positive relationship between BMI and plasma CLEC3B concentration (r = 0.492, *p* = 0.0147; *n* = 24) (Fig. 1e). In an exploratory multivariable linear regression model adjusted for age and PMOP status, BMI remained positively associated with plasma CLEC3B concentration (B = 0.587 ng/mL per kg/m^2^, 95% CI: 0.116 to 1.059, *p* = 0.017) [Supplementary Table S4]. PMOP status was also positively associated with plasma CLEC3B concentration (B = 3.026 ng/mL, 95% CI: 0.274 to 5.778, *p* = 0.033), whereas age was not significantly associated with plasma CLEC3B concentration (*p* = 0.844). Thus, increased circulating CLEC3B appears to characterize the combined high-BMI and PMOP context rather than PMOP alone.

We next asked whether estrogen-deficient mice showed a similar adipose-related pattern. Tissue profiling showed that *Clec3b* mRNA was most abundantly expressed in white adipose tissue (Fig. 1f). Ovariectomized (OVX) mice exhibited an increased gWAT-to-body-weight ratio compared with Sham controls (Supplementary Fig. 1a). Consistent with this adipose-enriched pattern, immunofluorescence staining revealed increased CLEC3B signal in gonadal white adipose tissue from OVX mice relative to Sham controls (Fig. 1g). In contrast, immunohistochemical analysis showed reduced CLEC3B expression in femoral bone tissue from OVX mice (Supplementary Fig. 1b). To determine whether estradiol directly regulates adipocytic CLEC3B expression, differentiated 3T3-L1 adipocytes were treated with increasing concentrations of estradiol. Estradiol reduced *Clec3b* mRNA expression in a dose-dependent manner (Fig. 1h) and decreased CLEC3B protein abundance (Fig. 1i). Collectively, these data identify CLEC3B as an adipose-enriched circulating factor preferentially associated with high-BMI PMOP and suggest that adipocytic CLEC3B expression is negatively regulated by estradiol.

### Adipose-targeted *Clec3b* overexpression promotes trabecular bone accrual in mice

**Fig. 2:**
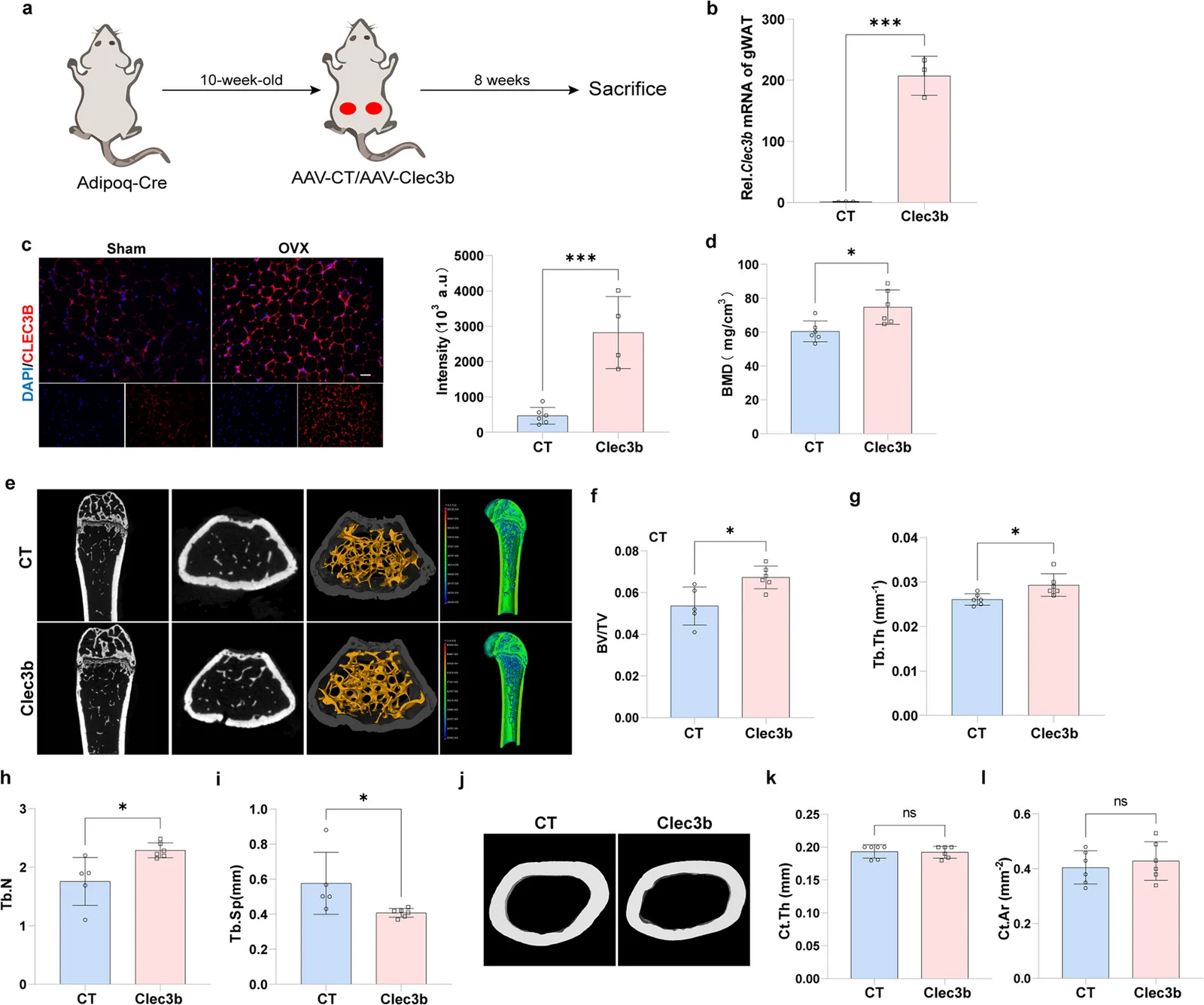
Adipose-targeted *Clec3b* overexpression promotes trabecular bone accrual in mice. **a** Schematic of AAV-mediated adipose-targeted overexpression of *Clec3b* in Adipoq-Cre mice for 8 weeks. **b** qPCR validation of *Clec3b* mRNA expression in gWAT (n = 3 per group). **c** Immunofluorescence of gWAT showing enhanced CLEC3B protein in AAV-Clec3b mice (n = 6 per group). Scale bar = 100 μm. **d** Quantitative analysis of bone mineral density (BMD) in the distal femur. **e–i** Representative micro-CT images and quantitative analysis of distal femoral trabecular bone showing increased bone volume fraction (BV/TV), trabecular thickness (Tb.Th), and trabecular number (Tb.N), together with decreased trabecular separation (Tb.Sp) (n = 6 per group). **j** Representative cortical bone images. **k, l** Quantitative analysis of cortical thickness (Ct.Th) and cortical area (Ct.Ar) (*n* = 6 per group). Data are presented as mean ± SD; *p < 0.05, **p < 0.01, ***p < 0.001, ****p < 0.0001; *ns*, not significant. Statistical analyses were performed using two-tailed Student’s t-test or one-way ANOVA followed by Tukey’s post hoc test

Given the adipose enrichment of CLEC3B and its preferential elevation in high-BMI PMOP, we tested whether raising adipose-derived *Clec3b* could alter skeletal homeostasis *in vivo*. To this end, we established an adipose-targeted gain-of-function model by delivering AAV-Clec3b to Adipoq-Cre mice (Fig. 2a). Successful overexpression was confirmed in gonadal white adipose tissue (gWAT), where *Clec3b* mRNA levels were markedly increased in the AAV-Clec3b group compared with controls (Fig. 2b). Consistently, immunofluorescence staining demonstrated a clear increase in CLEC3B protein abundance in gWAT after AAV delivery (Fig. 2c). Moreover, immunofluorescence analysis across multiple tissues confirmed that the increase in CLEC3B signal was predominantly detected in white adipose tissue, supporting the predominant adipose enrichment of the AAV-mediated gain-of-function model (Supplementary Fig. 2a).

We next evaluated the skeletal phenotype using high-resolution micro-CT of the distal femur. AAV-Clec3b mice exhibited significantly increased bone mineral density (BMD) compared with control mice (Fig. 2d). Three-dimensional reconstructions and microarchitectural measurements further indicated expansion of the trabecular compartment, including higher bone volume fraction (BV/TV), trabecular thickness (Tb.Th), and trabecular number (Tb.N), with lower trabecular separation (Tb.Sp) (Fig. 2e–i). In contrast, cortical bone architecture was not significantly altered, as cortical thickness and cortical area remained comparable between groups (Fig. 2j–l). Collectively, these findings indicate that adipose-targeted *Clec3b* overexpression preferentially promotes trabecular bone accrual *in vivo*.

### Adipose-targeted *Clec3b* overexpression preferentially enhances osteoblast-associated outcomes without markedly altering osteoclast-related parameters *in vivo*

**Fig. 3:**
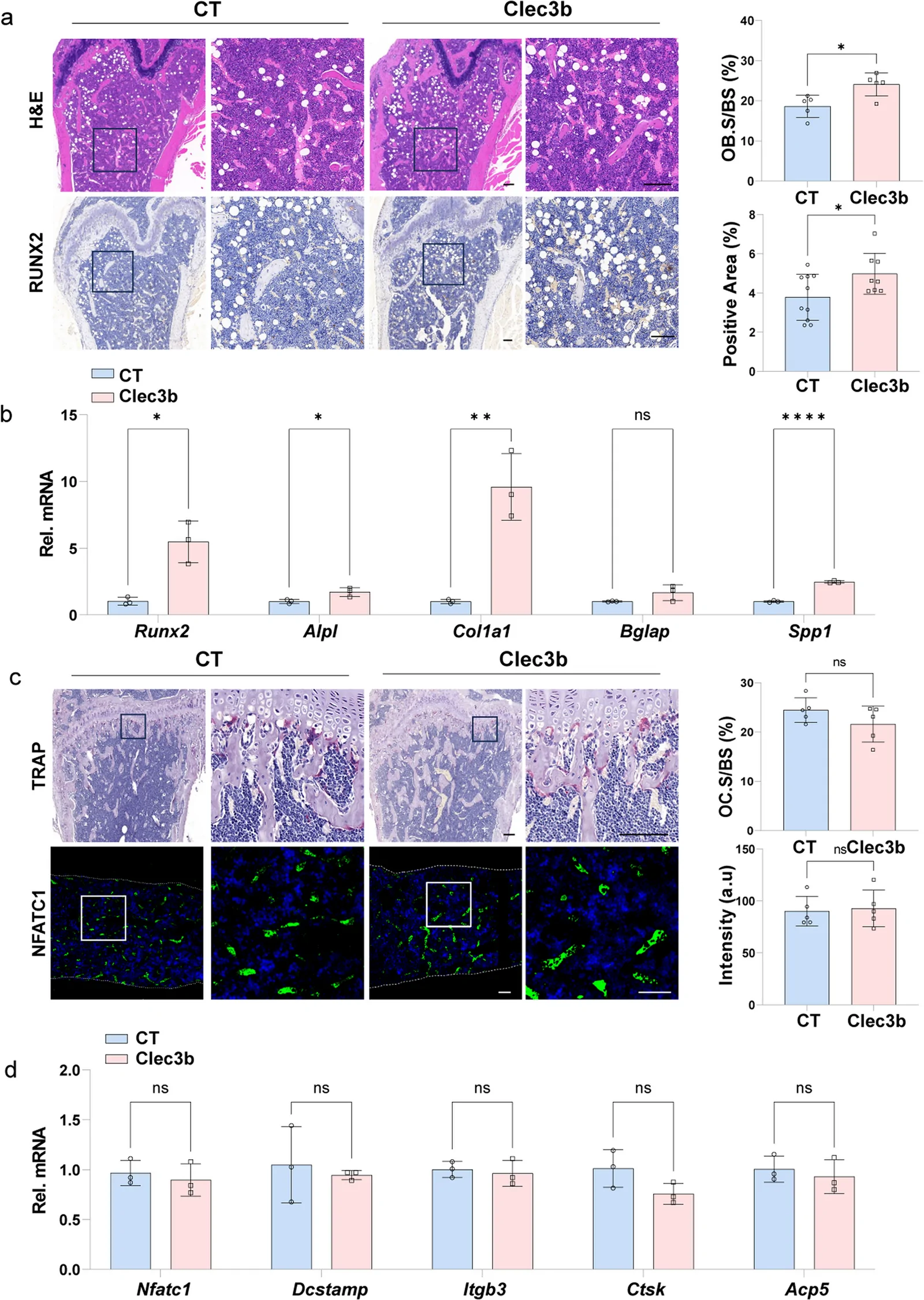
Adipose-targeted *Clec3b* overexpression preferentially enhances osteoblast-associated outcomes without markedly altering osteoclast-related parameters in vivo. **a** Representative H&E and RUNX2 staining of distal femur; quantification of osteoblast surface (Ob.S/BS) and RUNX2-positive area (*n* = 5 per group). Scale bar = 100 μm. **b** mRNA expression of osteogenic markers (*Runx2*, *Alpl*, *Col1a1*, *Bglap*, *Spp1*) in femoral bone samples (*n* = 3 per group). **c** TRAP staining and NFATC1 immunofluorescence of distal femoral sections showing osteoclast-related parameters (*n* = 5 per group). Scale bar = 100 μm. **d** Osteoclast-related genes (*Nfatc1*, *Dcstamp*, *Itgb3*, *Ctsk*, *Acp5*) expression in bone tissue (n = 3 per group). Data are presented as mean ± SD; *p < 0.05, **p < 0.01, ***p < 0.001, ****p < 0.0001; *ns*, not significant. Statistical analyses were performed using two-tailed Student’s t-test or one-way ANOVA followed by Tukey’s post hoc test

Having observed a marked trabecular bone phenotype in mice with adipose-targeted *Clec3b* overexpression, we then asked which cellular compartment primarily accounted for this effect. Histological examination of distal femoral sections showed greater trabecular bone area and increased osteoblast surface along trabeculae in the overexpression group (Fig. 3a). Consistently, immunohistochemical staining demonstrated an increase in RUNX2-positive area in the distal femur after adipose-targeted *Clec3b* overexpression (Fig. 3a). At the molecular level, qPCR analysis of femoral bone samples showed increased expression of *Runx2*, *Alpl*, *Col1a1*, *Spp1*, whereas *Bglap* expression was not significantly altered (Fig. 3b). Together, these findings indicate that adipose-targeted *Clec3b* overexpression is associated with enhanced osteoblast-lineage activity *in vivo*.

By contrast, osteoclast-related readouts remained largely unchanged. TRAP staining and NFATC1 immunofluorescence of distal femoral sections showed no significant differences between AAV-CT and AAV-Clec3b mice (Fig. 3c). In line with these histological observations, expression of osteoclast-related genes in bone tissue, including *Nfatc1*, *Dcstamp*, *Itgb3*, *Ctsk*, *Acp5*, remained largely unchanged (Fig. 3d). Collectively, these results suggest that the increase in trabecular bone mass induced by adipose-targeted *Clec3b* overexpression is predominantly associated with enhanced osteoblast-related responses rather than with major alterations in the osteoclast compartment under the present experimental conditions.

### Conditioned medium from *Clec3b*-overexpressing gWAT promotes osteogenic differentiation of BMSCs without markedly altering osteoclast-related outcomes

**Fig. 4:**
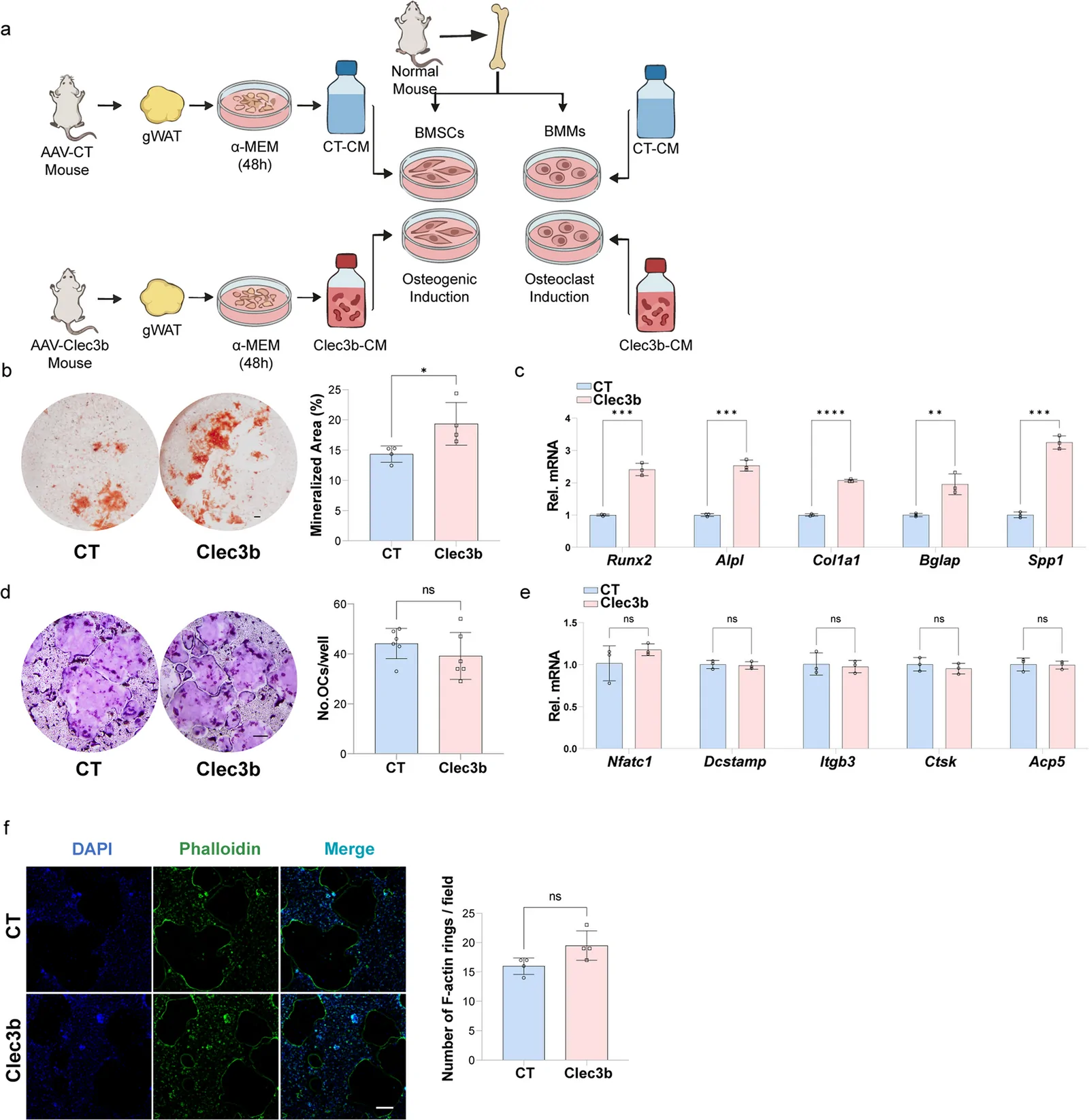
Conditioned medium from *Clec3b*-overexpressing adipose tissue promotes osteogenic differentiation of BMSCs without markedly altering osteoclast-related outcomes. **a** Experimental design showing collection of conditioned medium (CM) from gWAT of AAV-CT or AAV-Clec3b mice and its application to BMSCs and BMMs. **b** Alizarin Red S staining and quantification of mineralized matrix formation in BMSCs treated with CT-CM (CT) or Clec3b-CM (Clec3b) (*n* = 4 per group). Scale bar = 200 μm. **c** Osteogenic-related gene (*Runx2*, *Alpl*, *Col1a1*, *Bglap*, *Spp1*) expression in BMSCs treated with CT-CM (CT) or Clec3b-CM (Clec3b) (*n* = 3 per group). **d** TRAP staining of BMMs shows no significant difference in osteoclast differentiation. Scale bar = 100 μm. **e** Osteoclast-related genes (*Nfatc1*, *Dcstamp*, *Itgb3*, *Ctsk*, *Acp5*) expression after CM treatment (*n* = 3 per group). **f** Phalloidin staining of actin rings in BMMs, confirming no significant structural changes. Scale bar = 100 μm. Data are presented as mean ± SD; *p < 0.05, **p < 0.01, ***p < 0.001; *ns*, not significant. Statistical analyses were performed using two-tailed Student’s t-test or one-way ANOVA followed by Tukey’s post hoc test

Because the *in vivo* phenotype was mainly osteoblast-associated, we examined whether adipose *Clec3b* overexpression produced transferable paracrine cues that favor osteogenic differentiation. Conditioned medium (CM) was collected from gonadal white adipose tissue (gWAT) of AAV-CT and AAV-Clec3b mice and applied to BMSCs and BMMs under osteogenic or osteoclastogenic conditions, respectively (Fig. 4a). BMSCs treated with CM from *Clec3b*-overexpressing gWAT displayed a marked increase in mineralized matrix formation, as shown by Alizarin Red S staining (Fig. 4b). In parallel, qPCR analysis demonstrated that *Clec3b*-enriched CM significantly upregulated the expression of osteogenic genes, including *Runx2*, *Alpl*, *Col1a1*, *Bglap*, *and Spp1* (Fig. 4c). These data suggest that the secretome from *Clec3b*-overexpressing adipose tissue favors osteogenic differentiation of mesenchymal progenitors.

We next evaluated whether the same CM altered osteoclast-related outcomes. TRAP staining showed no significant difference in the number of TRAP-positive osteoclasts formed from BMMs treated with control or *Clec3b*-enriched gWAT-CM (Fig. 4d). Similarly, the expression of osteoclast-related genes, including *Nfatc1, Dcstamp*, *Itgb3*, *Ctsk*, *Acp5*, remained largely unchanged after CM treatment (Fig. 4e). Consistently, phalloidin staining revealed no apparent structural differences in actin ring formation (Fig. 4f). Taken together, these data support a preferential pro-osteogenic paracrine effect associated with adipose *Clec3b* overexpression, without evidence of marked alterations in osteoclast differentiation under the present experimental conditions.

### *Clec3b*-overexpressing adipocyte-conditioned medium promotes osteogenic differentiation and is associated with mitochondrial remodeling in osteoblasts

**Fig. 5:**
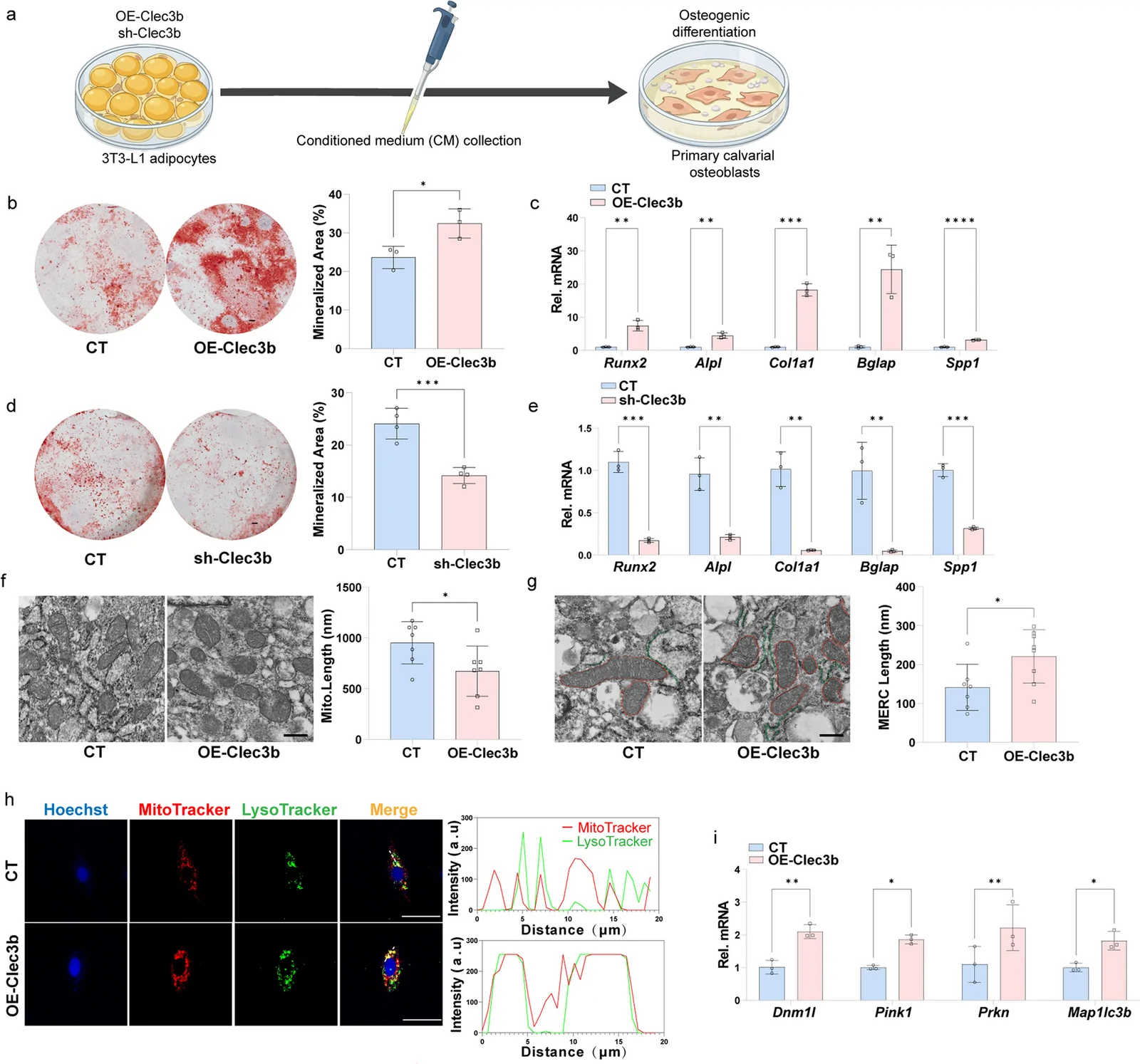
*Clec3b*-enriched 3T3-L1 adipocyte-conditioned medium promotes osteogenic differentiation and is associated with mitochondrial remodeling in vitro. **a** CM from 3T3-L1 adipocytes with *Clec3b* overexpression (OE) or knockdown (sh) applied to primary calvarial osteoblasts. **b**, **d** Alizarin Red S staining and quantification of the mineralized area (*n* = 4 per group). Scale bar = 200 μm. **c, e** Osteogenic-related gene expression *(Runx2*, *Alpl*, *Col1a1*, *Bglap*, *Spp1)* after OE-Clec3b or sh-Clec3b CM treatment (*n* = 3 per group). **f** TEM images showing mitochondrial morphology; mitochondrial length quantified (*n* = 7 per group). Scale bar = 500 nm. **g** Quantification of MERC length from TEM images (*n* = 7 per group). Scale bar = 500 nm. **h** Mitochondria–lysosome overlap visualized by MitoTracker and LysoTracker. White dashed lines indicate line-scan analysis regions. Scale bar = 50 μm.** i** Mitochondrial dynamics- and quality-control-related genes *(Dnm1l*, *Pink1*, *Prkn*, *Map1lc3b)* expression after CM treatment (*n* = 3 per group). Data are presented as mean ± SD; *p < 0.05, **p < 0.01, ***p < 0.001, ****p < 0.0001; *ns*, not significant. Statistical analyses were performed using two-tailed Student’s t-test or one-way ANOVA followed by Tukey’s post hoc test

After observing preferential osteoblast-related effects *in vivo*, we used a reductionist adipocyte–osteoblast conditioned-medium system to assess paracrine effects on osteoblast function. 3T3-L1 adipocytes with *Clec3b* overexpression or knockdown were generated and validated (Supplementary Fig. 3a), and the corresponding conditioned media (CM) were applied to primary calvarial osteoblasts (Fig. 5a). CM from *Clec3b*-overexpressing adipocytes significantly increased mineralized matrix formation and upregulated the osteogenic and matrix-associated genes *Runx2*, *Alpl*, *Col1a1*, *Bglap*, and *Spp1* (Fig. 5b, c). Conversely, CM from *Clec3b*-silenced adipocytes reduced mineralized matrix formation and downregulated these markers (Fig. 5d, e). In parallel, CM derived from 3T3-L1 adipocytes with Clec3b overexpression or knockdown did not significantly affect TRAP-positive osteoclast generation or the expression of osteoclast-associated genes in bone marrow-derived macrophages (BMMs) (Supplementary Fig. 3b–f). These findings support a preferential paracrine effect of adipocytic *Clec3b* on osteoblast differentiation.

We then assessed whether this osteogenic response was accompanied by changes in mitochondrial organization. Transmission electron microscopy showed that osteoblasts exposed to *Clec3b*-enriched CM exhibited shorter mitochondria than control cells (Fig. 5f). Under the same condition, quantitative analysis revealed an increase in mitochondria–endoplasmic reticulum contact (MERC) length (Fig. 5g). MitoTracker Red and LysoTracker Green staining, together with line-scan fluorescence profiles, further suggested greater mitochondria–lysosome overlap in osteoblasts treated with *Clec3b*-enriched CM [Fig. 5h]. Consistently, qPCR analysis revealed upregulated expression of genes related to mitochondrial dynamics and quality-control-related processes, including *Dnm1l*, *Pink1*, *Prkn*, and *Map1lc3b* (Fig. 5i). Thus, *Clec3b*-enriched adipocyte CM enhanced osteoblast differentiation and coincided with changes in mitochondrial organization and quality-control-related markers.

### Adipocytic *Clec3b* suppresses *Igfbp4* and is associated with osteogenic and mitochondrial responses in osteoblasts

**Fig. 6:**
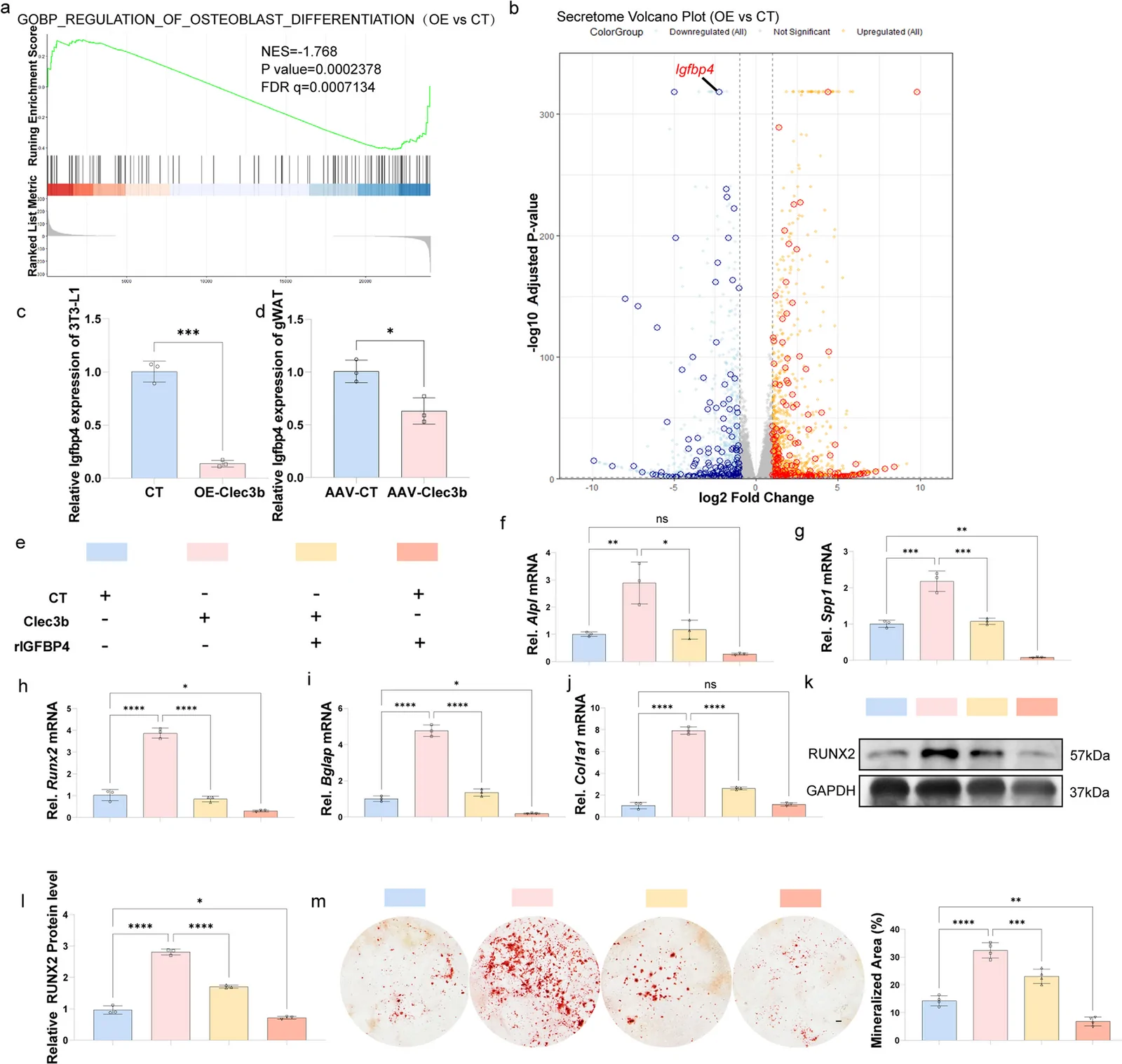
CLEC3B enhances the pro-osteogenic effect of 3T3-L1 adipocyte-conditioned medium on primary calvarial osteoblasts by suppressing adipocytic IGFBP4 expression. **a** GSEA plot showing negative enrichment of the osteoblast differentiation gene set in RNA-seq of OE-Clec3b versus CT 3T3-L1 cells. Genes were ranked by log2 fold change (log2FC). **b** Volcano plot of differentially expressed genes in OE-Clec3b versus CT 3T3-L1 adipocytes. Genes meeting the significance criteria of adjusted *p* < 0.05 and *|log2 fold change|*≥ 1 are highlighted. Open circles indicate genes annotated as secreted proteins based on Gene Ontology (GO) terms. *Igfbp4* is highlighted as a significantly downregulated secretome-associated candidate. **c, d** qPCR validation of *Igfbp4* expression in 3T3-L1 cells (**c**) and gWAT from AAV-CT and AAV-Clec3b mice (**d**) (*n* = 3 per group). **e** Rescue experiments in primary calvarial osteoblasts treated with control CM, *Clec3b*-enriched CM, *Clec3b*-enriched CM supplemented with recombinant IGFBP4, or control CM supplemented with recombinant IGFBP4. f–j Osteogenic-related gene *(Alpl*, *Spp1*, *Runx2*, *Bglap*, *Col1a1)* expression (*n* = 3 per group). **k, l** Representative western blot images (**k**) and densitometric quantification (**l**) of RUNX2 protein expression in primary calvarial osteoblasts under the indicated rescue conditions. GAPDH served as a loading control (*n* = 3 per group). **m** Representative Alizarin Red S staining showing partial attenuation by rIGFBP4 (*n* = 4 per group). Scale bar = 200 μm. Data are presented as mean ± SD; *p < 0.05, **p < 0.01, ***p < 0.001, ****p < 0.0001; *ns*, not significant. Statistical analyses were performed using two-tailed Student’s t-test or one-way ANOVA followed by Tukey’s post hoc test

Because *Clec3b*-enriched adipocyte-conditioned medium (CM) preferentially promoted osteogenesis, we examined whether adipocytic *Clec3b* altered the adipocyte secretory program. RNA sequencing of control and *Clec3b*-overexpressing 3T3-L1 adipocytes revealed negative enrichment of the osteoblast differentiation gene set in the overexpression group (Fig. 6a). Focusing on secretome-associated genes, volcano plot analysis identified *Igfbp4* as one of the most prominently downregulated candidates in the *Clec3b*-overexpression group (Fig. 6b). Because IGFBP4 is a secreted regulator of bone homeostasis, these findings suggested that suppression of adipocytic *Igfbp4* may contribute to the pro-osteogenic paracrine effects associated with *Clec3b* overexpression.

The qPCR analysis confirmed that *Igfbp4* expression was significantly reduced in *Clec3b*-overexpressing 3T3-L1 adipocytes (Fig. 6c). A similar decrease was observed in gWAT from mice with adipose-targeted *Clec3b* overexpression (Fig. 6d), indicating that suppression of adipocytic *Igfbp4* is a consistent response to *Clec3b* elevation across experimental systems. We therefore performed rescue experiments in primary calvarial osteoblasts using control CM, *Clec3b*-enriched CM, *Clec3b*-enriched CM supplemented with recombinant IGFBP4 (rIGFBP4), or control CM supplemented with rIGFBP4 (Fig. 6e). *Clec3b*-enriched CM increased the expression of osteogenic and matrix-associated genes, including *Alpl*, *Spp1*, *Runx2*, *Bglap*, and *Col1a1*, whereas rIGFBP4 supplementation attenuated these responses (Fig. 6f–j). Consistently, RUNX2 protein abundance was increased by *Clec3b*-enriched CM and reduced after rIGFBP4 supplementation (Fig. 6k, l). ALP staining and activity assays further showed that *Clec3b*-enriched CM enhanced osteogenic differentiation, whereas rIGFBP4 partially attenuated this effect (Supplementary Fig. 5). Alizarin Red S staining further showed enhanced mineralized matrix formation in the *Clec3b*-enriched CM group, which was partially attenuated by rIGFBP4 (Fig. 6m). These findings place IGFBP4 as an inhibitory downstream component of the *Clec3b*-associated osteogenic response.

**Fig. 7:**
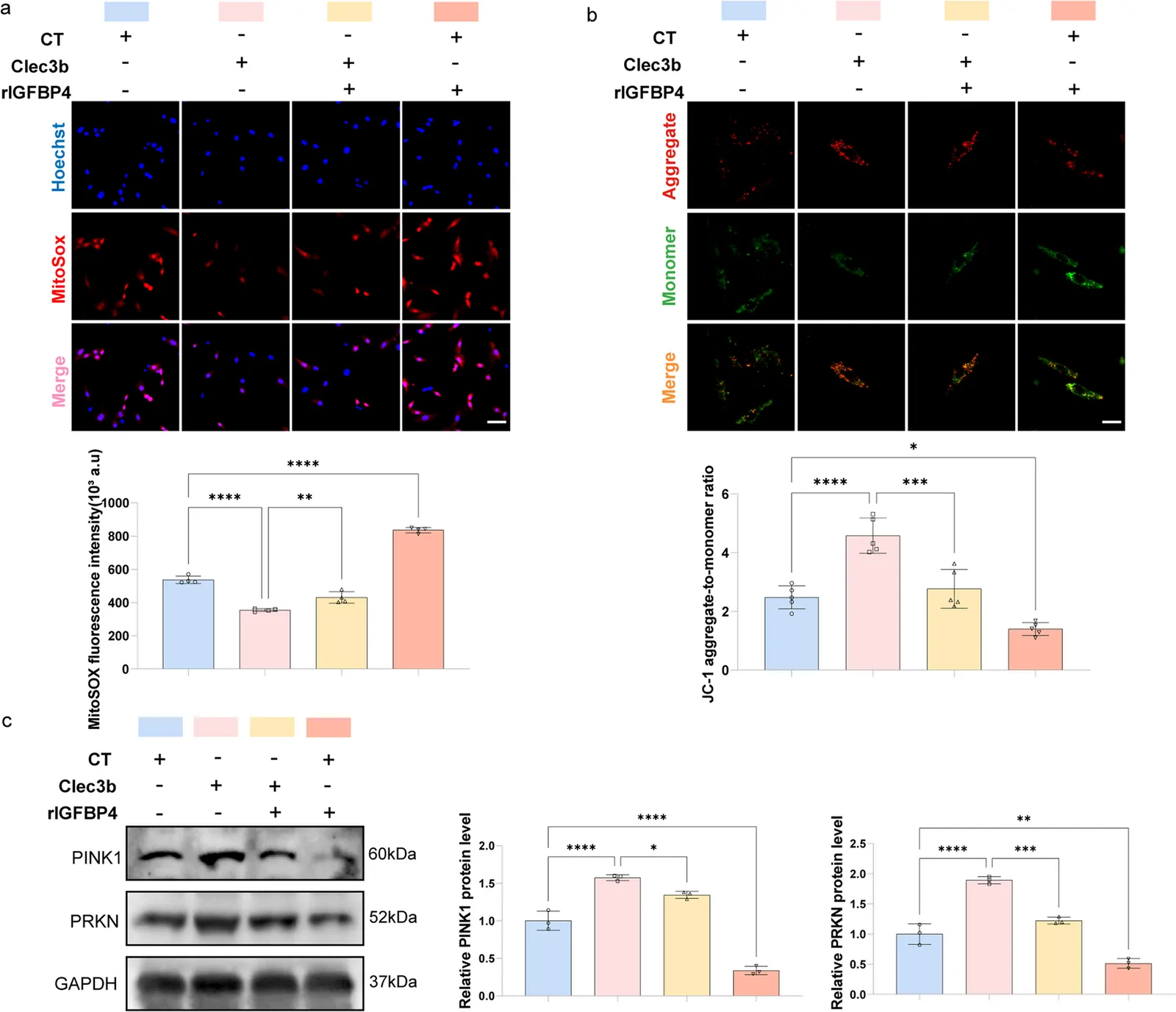
rIGFBP4 attenuates *Clec3b*-enriched adipocyte CM-induced improvements in mitochondrial redox status, membrane potential, and mitochondrial quality-control-related signaling in primary calvarial osteoblasts. **a** Representative MitoSOX staining images and quantification of mitochondrial superoxide-related fluorescence intensity in primary calvarial osteoblasts treated with conditioned medium (CM) under the indicated conditions (*n* = 5 per group). Nuclei were counterstained with Hoechst. Scale bar = 100 μm. **b** Representative JC-1 staining images and quantification of the aggregate-to-monomer fluorescence ratio (*n* = 5 per group). Red fluorescence indicates JC-1 aggregates, reflecting relatively high mitochondrial membrane potential, whereas green fluorescence indicates JC-1 monomers, reflecting relatively low mitochondrial membrane potential. Scale bar = 25 μm. **c** Western blot analysis of PINK1 and PRKN protein levels. GAPDH served as a loading control. (*n* = 3 per group). Data are presented as mean ± SD; *p < 0.05, **p < 0.01, ***p < 0.001, ****p < 0.0001; *ns*, not significant. Statistical analyses were performed using one-way ANOVA followed by Tukey’s post hoc test

We further applied the rescue design to evaluate mitochondrial adaptation in osteoblasts. MitoSOX staining showed reduced mitochondrial superoxide-related fluorescence in cells exposed to *Clec3b*-enriched CM, whereas rIGFBP4 partially attenuated this change (Fig. 7a). JC-1 staining showed an increased aggregate-to-monomer ratio after *Clec3b*-enriched CM treatment, consistent with improved mitochondrial membrane potential; this effect was attenuated by rIGFBP4 (Fig. 7b). Western blotting further showed increased PINK1 and PRKN protein levels under *Clec3b*-enriched CM conditions, with partial attenuation after rIGFBP4 supplementation (Fig. 7c). DCFH-DA and DHE staining provided additional evidence of corresponding changes in intracellular oxidative fluorescence (Supplementary Fig. 4a, b). CQ-based LC3B analysis further supported altered autophagy-related LC3B dynamics under CLEC3B–IGFBP4-related conditions (Supplementary Fig. 4c).

Overall, these data suggest that reduced adipocytic IGFBP4 contributes in part to the osteogenic effect of *Clec3b*-enriched CM and parallels improvements in mitochondrial redox status, membrane potential, and quality-control-related signaling in osteoblasts.

## Discussion

In this study, CLEC3B emerged as an adipose-enriched circulating factor preferentially increased in the high-BMI postmenopausal osteoporosis (PMOP) subgroup. By combining adipose-targeted gain-of-function models with ex vivo and *in vitro* conditioned-medium systems, we found that adipose-associated *Clec3b* elevation increased trabecular bone accrual mainly by enhancing osteoblast activity rather than by strongly suppressing osteoclastogenesis. Our data further support a model in which *Clec3b* remodels the adipocyte secretome, suppresses the inhibitory factor IGFBP4, and is associated with improved mitochondrial redox status and mitochondrial quality-control-related responses in osteoblast-lineage cells (Li et al. 2023). Collectively, these findings support a previously underrecognized adipose-to-bone regulatory axis and broaden current understanding of how metabolic signals may influence skeletal homeostasis in PMOP.

The influence of obesity on the skeleton is context dependent and not uniformly protective (Ali et al. 2022). Although higher body weight has traditionally been considered beneficial to bone because of increased mechanical loading, body mass alone does not fully reflect the skeletal consequences of excess adiposity. Adipose-tissue distribution, inflammatory status, and secretory function may influence bone remodeling independently of mechanical loading. In particular, marrow adiposity has been associated with impaired osteogenesis, while obesity-related metabolic stress can promote adipose-tissue inflammation and secretome reprogramming (Veldhuis-Vlug and Rosen 2018; Bland et al. 2022). Altered release of adipokines, cytokines, lipotoxic mediators, and extracellular vesicles may subsequently affect osteoblast-lineage cells (Musso et al. 2013; Zhang et al. 2021; Zhu et al. 2021; Veldhuis-Vlug and Rosen 2018). These observations indicate that obesity-related skeletal outcomes may reflect a balance between favorable loading effects and adverse adipose-derived metabolic signals. In this context, the preferential elevation of CLEC3B in the high-BMI PMOP subgroup supports the possibility that CLEC3B represents a metabolically responsive adipose-enriched mediator rather than a nonspecific correlate of skeletal aging. A key feature of our study is the predominance of osteoblast-directed, rather than osteoclast-directed, effects associated with adipose-derived CLEC3B. *In vivo*, adipose-targeted *Clec3b* overexpression increased trabecular bone mass together with osteoblast-associated histological and transcriptional readouts, whereas osteoclast-related indices showed no marked change. This pattern was recapitulated *in vitro* and ex vivo, where conditioned media derived from *Clec3b*-enriched adipose tissue or adipocytes enhanced mineralization and osteogenic gene expression with limited effect on osteoclast differentiation or actin-ring formation. This finding is biologically plausible because the adipocyte secretome can preferentially influence osteoblast-lineage commitment and proliferation (Zhang et al. 2021). Marrow adiposity has likewise been associated with impaired osteogenesis and reduced bone-forming capacity in metabolically unfavorable skeletal environments (Veldhuis-Vlug and Rosen 2017). In PMOP, where defective bone formation contributes to progressive trabecular deterioration, restoration of osteogenic capacity is pathophysiologically relevant (Gastel and Carmeliet 2021). It is also therapeutically meaningful because osteoblast bioenergetic programs are increasingly recognized as important determinants of bone-forming function (Shen et al. 2022). Together, these findings support the view that CLEC3B-associated adipose signaling preferentially promotes bone formation rather than broadly regulating both arms of bone remodeling.

Our mechanistic data also connect the CLEC3B–IGFBP4-related axis with adipocyte secretome remodeling and mitochondrial adaptation in osteoblasts. Transcriptomic analysis identified Igfbp4 as a prominent CLEC3B-responsive node in adipocytes. This result is consistent with the known role of IGFBP4 as a regulator of the bone microenvironment (Durham et al. 1994). *In vivo* and cell-based studies further indicate that elevated IGFBP4 restrains skeletal growth and osteogenic differentiation (Wu et al. 2017), while stromal-cell studies show that IGFBP4 can antagonize IGF-related responses in bone-associated microenvironments (Grellier et al. 1996). These data indicate that adipocytic *Clec3b* may shift the adipocyte secretome toward an osteogenesis-supportive profile, at least partly through reduced IGFBP4. Functionally, recombinant IGFBP4 reduced the osteogenic gene-expression response, RUNX2 protein abundance, ALP activity, and matrix mineralization induced by *Clec3b*-enriched conditioned medium, supporting IGFBP4 as an inhibitory mediator rather than only a transcriptomic candidate. This interpretation is consistent with evidence that relief of IGFBP4-mediated restraint, including through PAPP-A-dependent proteolysis, promotes osteoblast proliferation and bone formation (Qin et al. 2006).

At the organelle level, the CLEC3B–IGFBP4-related pathway was associated with mitochondrial adaptation in osteoblasts. Osteogenic commitment is known to depend on mitochondrial remodeling and metabolic rewiring (Li et al. 2017). More recent work has further emphasized that mitochondrial function is integral to osteoblast differentiation and lineage fate control (Suh and Lee 2024). In line with this framework, the reduced mitochondrial length, increased MERC length, and greater mitochondria–lysosome overlap observed in our study were consistent with active mitochondrial remodeling. Importantly, mitochondrial dynamics have been directly linked to mesenchymal lineage commitment and osteogenic differentiation (Forni et al. 2016). The accompanying reductions in mitochondrial superoxide-related and intracellular oxidative fluorescence signals were also consistent with the concept that oxidative stress constrains osteoblast function during skeletal aging (Nandy et al. 2024). Moreover, the increased JC-1 aggregate-to-monomer ratio, together with the upregulation of PINK1 and PRKN protein levels and CQ-based LC3B analysis, further supported the involvement of mitochondrial quality-control-related signaling and autophagy-related LC3B dynamics (Palikaras et al. 2015; Wang et al. 2020; Li et al. 2023). These observations, however, do not directly demonstrate increased mitophagic flux. Rather, they indicate that CLEC3B–IGFBP4-related paracrine signaling is associated with mitochondrial quality-control-related responses during osteogenic differentiation. More stringent mitochondria-specific flux assays and genetic perturbation approaches will be required to define the causal contribution of mitophagy.

These findings have several potential implications. The preferential increase in circulating CLEC3B in the high-BMI PMOP subgroup suggests that adipose-derived factors may contribute to the heterogeneity of PMOP rather than merely reflecting skeletal aging. This view is consistent with the concept that obesity-related skeletal risk cannot be inferred from body mass alone. More broadly, our results reinforce the concept that adipose–bone endocrine communication is a relevant regulatory component of metabolic bone disease (Liu et al. 2023). Within this framework, the CLEC3B–IGFBP4-related axis is of interest because it links adipocyte secretory remodeling to both osteogenic responses and mitochondrial adaptation in osteoblasts.

Several limitations should be considered. First, the clinical cohort was relatively small, and larger studies are needed to validate the association between circulating CLEC3B and PMOP, particularly in individuals in the high-BMI subgroup. Although the BMI–CLEC3B association remained detectable in an exploratory model adjusted for age and PMOP status, the limited cohort size precluded more extensive multivariable analyses and warrants validation in larger independent cohorts. Second, the *in vivo* experiments relied mainly on adipose-targeted *Clec3b* overexpression; adipose-targeted loss-of-function studies in OVX and obesity-related models will be required to establish the necessity of endogenous CLEC3B under pathological conditions. Third, although the mitochondrial morphology, oxidative fluorescence, membrane-potential, PINK1/PRKN, and CQ-based LC3B findings support mitochondrial quality-control-related responses, they do not directly establish mitophagic flux (Wang et al. 2020). More stringent mitochondria-specific flux assays and genetic perturbation approaches are therefore warranted. Finally, the partial attenuation observed with recombinant IGFBP4 suggests that other adipocyte-derived mediators may also participate in the secretome remodeling observed here (Huang et al. 2020). Collectively, our study supports a model in which adipose-derived CLEC3B promotes osteogenesis through a CLEC3B–IGFBP4-related paracrine axis associated with mitochondrial adaptation in PMOP.

## Supplementary Information

Below is the link to the electronic supplementary material.

Supplementary file1 (DOCX 2445 KB)

## Data Availability

The data supporting the findings of this study are available from the corresponding author upon reasonable request.
