# Supplementary material for: Adipose-derived CLEC3B promotes osteogenesis through a CLEC3B–IGFBP4-related paracrine axis associated with mitochondrial adaptation in postmenopausal osteoporosis
Source: Cell Biol Toxicol. 2026 Jul 3;42(1):106. doi: 10.1007/s10565-026-10223-x (PMC13574853; doi:10.1007/s10565-026-10223-x)
Supplement: Supplementary file 1 [file 10565_2026_10223_MOESM1_ESM.docx]

**Supplementary Methods**

**Viral manipulation of *Clec3b* in 3T3-L1 adipocytes and in vivo adipose-targeted AAV delivery**

For *Clec3b* overexpression, a retroviral vector carrying the full-length murine *Clec3b* coding sequence was obtained from Shandong Weizhen Biotechnology Co., Ltd. (Jinan, China). Retroviral particles were generated using Plat-E packaging cells. The corresponding empty retroviral vector was used as the control.

For *Clec3b* knockdown, lentiviral particles expressing a short hairpin RNA targeting murine *Clec3b* were obtained from Tsingke Biotechnology Co., Ltd. (Beijing, China). The target sequence was 5′-GCGATCAGTTGCCCTACATCT-3′, and the full shRNA sequence was 5′-gatcgGCGATCAGTTGCCCTACATCTCTCGAGAGATGTAGGGCAACTGATCGCTTTTTT-3′. A non-targeting shRNA lentiviral vector was used as the negative control.

For viral transduction, 3T3-L1 cells were exposed to the indicated viral particles under optimized transduction conditions. Puromycin selection was initiated 48 h after transduction and maintained for 2 days. *Clec3b* overexpression and knockdown efficiency were verified by quantitative real-time PCR before conditioned media were collected for subsequent experiments.

For in vivo adipose-targeted gain-of-function experiments, AAV9-CAG-DIO-Clec3b and the corresponding control AAV vector were obtained from Shandong Weizhen Biotechnology Co., Ltd. (Jinan, China). Viral particles were delivered by bilateral gWAT injection as described in the main Methods section.

**Supplementary Figure 1. OVX increases the gWAT-to-body-weight ratio but reduces CLEC3B expression in bone tissue. a** Gonadal white adipose tissue (gWAT) weight normalized to total body weight in Sham and OVX mice. **b** Representative immunohistochemical staining of CLEC3B in femoral bone sections from Sham and OVX mice. Quantification of the CLEC3B-positive area is shown on the right. Data are presented as mean ± SEM. ***p* < 0.01, *****p* < 0.0001. Scale bar = 100 μm.

**Supplementary Figure 2. Validation of AAV-mediated adipose-targeted *Clec3b* overexpression. a** Immunofluorescence images and quantification of CLEC3B expression in various tissues (liver, bone, heart, kidney, BAT, and WAT), showing predominant enrichment in WAT. Scale bar = 100 μm.**Supplementary Figure 3. 3T3-L1-derived conditioned medium does not markedly alter osteoclast differentiation. a** qPCR validation of *Clec3b* overexpression and knockdown in 3T3-L1 adipocytes. **b** Experimental scheme showing collection of conditioned medium (CM) from OE-Clec3b- or sh-Clec3b-transduced 3T3-L1 adipocytes and its application to BMMs for osteoclast differentiation. **c** TRAP staining of BMMs treated with CM collected from control or *Clec3b*-overexpressing 3T3-L1 adipocytes. Quantification of osteoclast numbers is shown on the right. Scale bar = 100 μm. **d** Relative mRNA expression of osteoclast-related genes (*Nfatc1*, *Dcstamp*, *Itgb3*, *Ctsk*, *Acp5*) in BMMs after treatment with CM from control or *Clec3b*-overexpressing 3T3-L1 adipocytes (n = 3 per group). **e** TRAP staining of BMMs treated with CM collected from control or *Clec3b*-silenced 3T3-L1 adipocytes. Quantification of osteoclast numbers is shown on the right (n = 5 per group). Scale bar = 100 μm. **f** Relative mRNA expression of osteoclast-related genes (*Nfatc1*, *Dcstamp*, *Itgb3*, *Ctsk*, *Acp5*) in BMMs after treatment with CM from control or *Clec3b*-silenced 3T3-L1 adipocytes (n = 3 per group). Data are presented as mean ± SD; ***p* < 0.01; *****p* < 0.0001; *ns*, not significant. Statistical analysis was performed using a two-tailed Student’s t-test.

**Supplementary Figure 4. CLEC3B–IGFBP4-related conditions are associated with intracellular oxidative fluorescence and autophagy-related LC3B dynamics in osteoblasts.** **a** Representative DCFH-DA staining images and quantification of intracellular oxidative fluorescence intensity in primary calvarial osteoblasts treated under the indicated conditioned-medium conditions (n = 4 per group). Nuclei were counterstained with Hoechst. Scale bar = 100 μm. **b** Representative DHE staining images and quantification of intracellular oxidative fluorescence intensity under the indicated treatment conditions (n = 4 per group). Nuclei were counterstained with DAPI. Scale bar = 100 μm. **c** Western blot analysis of LC3B-I and LC3B-II levels in primary calvarial osteoblasts treated under the indicated conditions, with or without chloroquine (CQ). GAPDH served as a loading control. Quantification of the LC3B-II/LC3B-I ratio and relative LC3B-II protein levels is shown on the right**.** Data are presented as mean ± SD. Statistical significance was determined using one-way ANOVA followed by Tukey’s post hoc test. **p* < 0.05, ***p* < 0.01, ****p* < 0.001, *****p* < 0.0001; ns, not significant.

**Supplementary Figure 5. rIGFBP4 partially attenuates Clec3b-enriched adipocyte CM-induced ALP activity in primary calvarial osteoblasts.** **a** Representative whole-well images of ALP staining in primary calvarial osteoblasts after 10 days of osteogenic induction under the indicated treatment conditions. Scale bar = 200 μm. **b** ALP activity was measured after 10 days of osteogenic induction, normalized to total protein content, and expressed as fold change relative to the CT group (n = 4 per group). Data are presented as mean ± SD. Statistical significance was determined using one-way ANOVA followed by Tukey’s post hoc test. **p* < 0.05, *****p* < 0.0001.

**Supplementary Table S1. Clinical characteristics and plasma CLEC3B ELISA data of postmenopausal women included in the final analysis**

*Abbreviations: BMI, body mass index; DXA, dual-energy X-ray absorptiometry; NA, not available. Plasma CLEC3B concentrations are presented in ng/mL.*

| **Original ID** | **Sex** | **Age** | **Height** **(cm)** | **Weight** **(kg)** | **BMI** **(kg/m²)** | **Lumbar spine T-score (L1–L4)** | **Femoral neck T-score** | **Total hip T-score** | **Admission diagnosis** | **Fasting** | **Plasma CLEC3B (ng/mL)** | **Clinical group** |
| --- | --- | --- | --- | --- | --- | --- | --- | --- | --- | --- | --- | --- |
| 1 | Female | 72 | 168 | 65 | 23.03 | -2.5 | NA | -1.5 | Lumbar spinal stenosis; L3 spondylolisthesis; lumbar disc herniation with radiculopathy | Yes | 7.410317 | Normal-BMI PMOP (PMOP) |
| 2 | Female | 60 | 170 | 65 | 22.49 | -2.5 | NA | -2 | Lumbar disc herniation with radiculopathy | Yes | 5.038475 | Normal-BMI PMOP (PMOP) |
| 3 | Female | 49 | 164 | 70 | 26.03 | 0.1 | NA | NA | Lumbar spinal stenosis; lumbar disc herniation | Yes | 8.607298 | High-BMI non-PMOP (High-BMI) |
| 10 | Female | 61 | 162 | 62 | 23.62 | -3.3 | NA | -1.7 | Lumbar spondylolisthesis | Yes | 2.771432 | Normal-BMI PMOP (PMOP) |
| 13 | Female | 76 | 155 | 60 | 24.97 | -2.7 | NA | -1.2 | Cervical spondylosis; lumbar spondylolisthesis; osteoporosis; emphysema | Yes | 6.078276 | Normal-BMI PMOP (PMOP) |
| 14 | Female | 70 | 163 | 58 | 21.83 | -0.9 | NA | NA | Lumbar disc herniation | Yes | 7.013766 | Normal-BMI non-PMOP (Normal) |
| 17 | Female | 52 | 157 | 63 | 25.56 | -1.4 | -1.6 | -0.9 | Lumbar spinal stenosis | Yes | 5.520305 | High-BMI non-PMOP (High-BMI) |
| 18 | Female | 60 | 150 | 65 | 28.89 | -2.7 | NA | NA | Lumbar disc herniation; old fracture | Yes | 12.10979 | High-BMI PMOP |
| 19 | Female | 46 | 165 | 54 | 19.83 | -1.1 | NA | NA | Lumbar disc herniation | Yes | 4.899411 | Normal-BMI non-PMOP (Normal) |
| 20 | Female | 65 | 167 | 63 | 22.59 | -3.1 | NA | NA | Scoliosis; lumbar spinal stenosis; osteoporosis | Yes | 8.503806 | Normal-BMI PMOP (PMOP) |
| 21 | Female | 62 | 153 | 63 | 26.91 | -3.5 | NA | -0.7 | Lumbar fracture; osteoporosis | Yes | 11.31427 | High-BMI PMOP |
| 22 | Female | 58 | 160 | 55 | 21.48 | -2.8 | NA | NA | Lumbar disc herniation; degenerative scoliosis | Yes | 7.973499 | Normal-BMI PMOP (PMOP) |
| 24 | Female | 66 | 160 | 59 | 23.05 | -0.9 | NA | -1.2 | Lumbar disc herniation; lumbar spinal stenosis | Yes | 4.893654 | Normal-BMI non-PMOP (Normal) |
| 27 | Female | 59 | 165 | 77 | 28.28 | -1.1 | 0.2 | 0.2 | Lumbar disc herniation with radicular symptoms; scoliosis | Yes | 6.456969 | High-BMI non-PMOP (High-BMI) |
| 31 | Female | 58 | 165 | 66 | 24.24 | -0.4 | NA | NA | Mixed cervical spondylosis | Yes | 7.286192 | Normal-BMI non-PMOP (Normal) |
| 32 | Female | 47 | 163 | 70 | 26.35 | 0.2 | NA | NA | Lumbar disc herniation with radicular symptoms; lumbar spinal stenosis | Yes | 6.359721 | High-BMI non-PMOP (High-BMI) |
| 33 | Female | 60 | 155 | 61 | 25.39 | -2.5 | NA | -1.3 | Lumbar disc herniation with radiculopathy; osteoporosis | Yes | 15.0589 | High-BMI PMOP |
| 36 | Female | 54 | 158 | 65 | 26.04 | -1.4 | -0.7 | 0.2 | Vertebral fracture | Yes | 5.939686 | High-BMI non-PMOP (High-BMI) |
| 37 | Female | 66 | 160 | 70 | 27.34 | -3.5 | NA | NA | Lumbar spinal stenosis | Yes | 14.26334 | High-BMI PMOP |
| 38 | Female | 72 | 150 | 60 | 26.67 | -2.9 | NA | -0.8 | Lumbar spinal stenosis | Yes | 11.05208 | High-BMI PMOP |
| 39 | Female | 49 | 165 | 63 | 23.14 | 2 | 1.4 | 2 | Lumbar disc herniation | Yes | 5.927158 | Normal-BMI non-PMOP (Normal) |
| 46 | Female | 47 | 160 | 58 | 22.66 | -0.7 | -0.8 | -0.7 | Lumbar disc herniation with radiculopathy | Yes | 5.556763 | Normal-BMI non-PMOP (Normal) |
| 51 | Female | 53 | 165 | 62 | 22.77 | 1.1 | NA | NA | Mixed cervical spondylosis | Yes | 5.538521 | Normal-BMI non-PMOP (Normal) |
| 52 | Female | 52 | 165 | 70 | 25.71 | 0.2 | NA | NA | Lumbar disc herniation with sciatica; right foot drop; arrhythmia | Yes | 4.392992 | High-BMI non-PMOP (High-BMI) |

**Supplementary Table S2. Primer sequences**

| Species | Target gene | Direction | Sequence (5'-3') |
| --- | --- | --- | --- |
| Mus | *Spp1* | F | GAGCGGGTGCTTGACAGAC |
| Mus | *Spp1* | R | ATGCGGCCTGCCATTTGTAT |
| Mus | *Clec3b* | F | ACGGAGATCACGACGCAAC |
| Mus | *Clec3b* | R | TTGTCGAACCACTTGCCGTT |
| Mus | *Igfbp4* | F | AGAAGCCCCTGCGTACATTG |
| Mus | *Igfbp4* | R | TTGTTGGGATGTTCGCTCTCA |
| Mus | *Actb* | F | CAGATGCCACTACAGCACG |
| Mus | *Actb* | R | CCTGCCGCTGCCATAGAAG |
| Mus | *Nfatc1* | F | ATACCTGGCTCGGTAACACC |
| Mus | *Nfatc1* | R | CATGCTCCAGTGCTGTCTTT |
| Mus | *Itgb3* | F | GATGACATCGAGCAGCTGAAAGAG |
| Mus | *Itgb3* | R | CCGGTCATGAATGGTGATGAGTAG |
| Mus | *Acp5* | F | ATGGGCGCTGACTTCATCAT |
| Mus | *Acp5* | R | GGTCTCCTGGAACCTCTTGT |
| Mus | *Dcstamp* | F | ACAAACAGTTCCAAAGCTTGC |
| Mus | *Dcstamp* | R | TCCTTGGGTTCCTTGCTTC |
| Mus | *Ctsk* | F | AGTAGCCACGCTTCCTATCC |
| Mus | *Ctsk* | R | CCATGGGTAGCAGCAGAAAC |
| Mus | *Runx2* | F | CGCCCCTCCCTGAACTCT |
| Mus | *Runx2* | R | TGCCTGCCTGGGATCTGTA |
| Mus | *Sp7* | F | GGAAAGGAGGCACAAAGAAGC |
| Mus | *Sp7* | R | CCCCTTAGGCACTAGGAGC |
| Mus | *Col1a1* | F | CGAAGGCAACAGTCGATTCA |
| Mus | *Col1a1* | R | CCCCAAGTTCCGGTGTGA |
| Mus | *Alpl* | F | ACACCAATGTAGCCAAGAATGTCA |
| Mus | *Alpl* | R | GATTCGGGCAGCGGTTACT |
| Mus | *Ibsp* | F | CCGGCCACGCTACTTTCTT |
| Mus | *Ibsp* | R | GGACTGGAAACCGTTTC |
| Mus | *Bglap* | F | CTGACAAAGCCTTCATGTCCAA |
| Mus | *Bglap* | R | GCGCCGGAGTCTGTTCACTA |
| Mus | *Dnm1l* | F | GCAACTGGAGAGGAATGCTG |
| Mus | *Dnm1l* | R | TGGAACTGGCACATCTAGCA |
| Mus | *Map1lc3b* | F | CCTTCTTCCTGCTGGTCAAC |
| Mus | *Map1lc3b* | R | AAGGTTTCTTGGGAGGCGTA |
| Mus | *Pink1* | F | CGAGCATCTTCTAGCCCTGA |
| Mus | *Pink1* | R | CTCACACTCCAGGTTAGCCA |
| Mus | *Prkn* | F | TGGAAAGCTCCGAGTTCAGT |
| Mus | *Prkn* | R | GTTGGGTGTGCTCCACATTT |

**Supplementary Table S3. Primary and secondary antibodies used in this study**

| **Target** | **Host species** | **Supplier** | **Catalog No.** | **Application** | **Dilution** | **Used in** |
| --- | --- | --- | --- | --- | --- | --- |
| CLEC3B | Rabbit | Abcam | ab108999 | IF, IHC, WB | IF/IHC: 1:200; WB: 1:2000 | Fig. 1g, i; Fig. 2c; Supplementary Fig. 1b; Supplementary Fig. 2 |
| RUNX2 | Rabbit | Abcam | ab192256 | WB/IHC | IHC: 1:200 WB: 1:1000 | Fig. 3a; Fig. 6k |
| NFATC1 | Rabbit | Abcam | ab25916 | IF | 1:200 | Fig. 3c |
| β-actin | Rabbit | Abcam | ab8227 | WB | 1:5000 | Fig. 1i |
| PINK1 | Rabbit | Proteintech | 23274-1-AP | WB | 1:2000 | Fig. 7c |
| PRKN (Parkin) | Rabbit | Proteintech | 14060-1-AP | WB | 1:2000 | Fig. 7c |
| LC3B | Rabbit | Proteintech | 81004-1-RR | WB | 1:3000 | Supplementary Fig. 4c |
| GAPDH | Rabbit | Proteintech | 10494-1-AP | WB | 1:5000 | Supplementary Fig. 4c; Fig. 6k; Fig. 7c |
| Alexa Fluor 488-conjugated secondary antibody | Goat anti-rabbit | Thermo Fisher Scientific | A11008 | IF | 1:500 | Bone and adipose IF staining |
| Alexa Fluor 555-conjugated secondary antibody | Donkey anti-rabbit | Abcam | ab150074 | IF | 1:500 | Bone and adipose IF staining |
| HRP-conjugated secondary antibody | Goat anti-rabbit | Proteintech | RGAR001 | WB | 1:3000 | WB |

**Supplementary Table S4. Exploratory multivariable linear regression analysis of factors associated with plasma CLEC3B concentration.**

| **Variable** | **Regression coefficient, B** | **Standard error** | **95% CI** | ***p* value** |
| --- | --- | --- | --- | --- |
| BMI, kg/m² | 0.587 | 0.226 | 0.116 to 1.059 | 0.017 |
| PMOP status, PMOP vs non-PMOP | 3.026 | 1.319 | 0.274 to 5.778 | 0.033 |
| Age, years | −0.015 | 0.077 | −0.177 to 0.146 | 0.844 |

Plasma CLEC3B concentration was entered as the dependent variable. PMOP status was coded as 0 for non-PMOP and 1 for PMOP. B, unstandardized regression coefficient; CI, confidence interval; BMI, body mass index; PMOP, postmenopausal osteoporosis. Model R² = 0.455; adjusted R² = 0.373; *F*(3, 20) = 5.558; model *p* = 0.0061.
